# Knowledge and attitudes towards E-cigarette use in Lebanon and their associated factors

**DOI:** 10.1186/s12889-020-8381-x

**Published:** 2020-02-28

**Authors:** Hanan Aghar, Nathalie El-Khoury, Mahasen Reda, Wissam Hamadeh, Hussein Krayem, Mohammad Mansour, Hawraa Raouf, Miran A. Jaffa

**Affiliations:** 1 Faculty of Medicine, American University of Beirut, P.O.Box 11-0236 Riad El-Solh, Beirut, 1107 2020 Lebanon; 2 Epidemiology and Population Health Department, Faculty of Health Sciences, American University of Beirut, P.O.Box 11-0236 Riad El-Solh, Beirut, 1107 2020 Lebanon

**Keywords:** Attitude, E-cigarettes, Electronic cigarettes, E-liquid, Knowledge, Perception, Smoking, Smoking cessation, Tobacco, Vaping

## Abstract

**Background:**

Despite the misconceptions regarding E-cigarettes (ECs), only a few studies have been conducted in the Middle East that focused on this topic. This study assesses the knowledge of and attitudes towards ECs in Lebanon, determines how these two measures are associated, and identifies the variables that explain each of these measures.

**Methods:**

A cross sectional study was conducted on a convenience sample of Lebanese pedestrians aged between 18 and 64 inclusive. A structured self-administered questionnaire comprising of knowledge and attitude scales, and questions on demographical, health and smoking characteristics was used.

**Results:**

Scores for attitudes and knowledge of ECs were summed and dichotomized using a 75% cutoff, above which the participant was considered to have a positive attitude and good knowledge. Among the 352 participants (56.6% males, 43.3% females, mean age 30.3, 46.2% smokers), 63.3% exhibited a lower level of EC knowledge. More than 50% erroneously thought that ECs are not associated with lung and bladder cancer or impair lung and heart function. 65% falsely thought that it is harmless and not addictive. As for attitude, 43.3, 53.9, and 44.3% thought that it is socially acceptable, helps in smoking cessation, and is a good replacement for cigarettes and an enjoyable recreational device respectively. Our results revealed an inverse correlation between attitude and knowledge scores (Spearman’s correlation = −.30, *p* < .001). Predictors of knowledge included health-related occupation (*p* = .010), regular exercise (*p* = .016), healthy diet (*p* = .026), EC use (p = .026), perception that ECs are not harmful (*p* = .001), and help in smoking cessation (*p* = .017). Predictors of attitude included EC use (*p* = .008), sex (*p* = .010), and knowledge that most ECs are addictive (*p* = .006), harmful (*p* = .014), and impair heart and lung function (*p* = .047).

**Conclusions:**

Our study revealed a gap in EC knowledge, especially among participants who displayed a positive attitude towards ECs. Hence, measures should be undertaken to regulate its use by instituting more stringent laws and holding nationwide awareness campaigns.

## Introduction

Electronic cigarettes or E-cigarettes (ECs) are vaping devices run on lithium ion batteries that have been marketed as a new method for smoking cessation. The major reasons for vaping include its usefulness in: 1- smoking cessation, 2- decreasing cigarette consumption, 3- abating tobacco craving, 4- mitigating harm when used as substitute for regular cigarettes, in addition to 5- being cheaper than ordinary cigarettes, and 6- having an enhanced taste and smell compared to ordinary cigarettes [1]. Although some brands of ECs do not contain nicotine [2], most deliver vapor containing nicotine (4–20 mg/ puff), along with flavors such as propylene glycol and glycerin [3]. When this study was conducted, EC use had still not been approved by the Food and Drug Administration (FDA). The delay in its approval could be because there is no reliable consensus as to whether EC is an effective smoking cessation aid that is safe and healthier than its tobacco counterparts with a comparatively lower or absent reliance and/or addiction potential [4].

Various studies assessing the effectiveness of ECs as smoking cessation aids presented conflicting findings. A systematic review showed that smokers using ECs as a smoking cessation method were less likely to quit smoking [5]. Another study documented a significant increase in the incidence of EC use among young people who have never smoked [3]. Nevertheless, other studies demonstrated its positive effect as a smoking cessation method. In this respect, it was shown that when tobacco smokers were encouraged to switch their consumption to ECs, there was a decrease in the number of tobacco cigarettes smoked per day and smokers became less dependent on cigarettes and more motivated to quit smoking [6]. There are also opposing views as to whether ECs are healthier alternatives to tobacco smoking. ECs were found to be less harmful than cigarettes as they do not combust, do not contain tobacco, and their vapor contains less toxic chemicals than tobacco [7]. It has also been shown that ECs were intended as a substitute for tobacco cigarettes among most smokers [8] who believed in its reduced risk for developing lung or oral cancers or heart disease compared to tobacco [9]. On the other hand, other studies have identified risk factors for the chronic use of ECs challenging the safety this smoking device was reputed to possess. While its use is less of a risk factor for cancer compared to cigarettes, the risk for developing long-term non-cancerous cardiovascular and pulmonary conditions is similar. This is important because most deaths caused by cigarettes are due to non-cancerous complications [10]. Furthermore, nicotine-containing ECs were found to cause chronic obstructive pulmonary disease (COPD) and morphological changes like an increase in immune cell reaction, lung tissue damage, and airway hypersensitivity [11]. Moreover, similarly to tobacco cigarettes, EC emissions were associated with both second-hand [12] and third-hand smoking [13].

The safety of the liquid in ECs, E-liquid (EL), is also questionable. Although not all ELs contain nicotine, most include a range of other potentially hazardous substances like formaldehyde, acetaldehyde, acrolein, propanal, nicotine, acetone, o-methyl-benzaldehyde, and carcinogenic nitrosamines, which were found to be toxic to cells [14]. In addition, it has been established that EL contains carcinogenic metals such as cadmium, chromium, nickel, lead, and manganese [15]. Flavorings in the EL, intended to appeal to consumers, also contain lung toxins like diacetyl and diketones [16]; the former of which is associated with bronchiolitis obliterans, commonly known as popcorn lung disease [17]. Toxicity is marked in all flavorings. However, fruit flavoring contributed more to cell mortality than tobacco flavoring [18]. Additionally, menthol caused a more prominent decrease in middle ear cell viability as well as perpetuated otitis media [19].

Given that ECs are relatively new devices, assessing individuals’ knowledge and attitude towards its harms and benefits has become a subject of research interest in specific regions in the world. For instance, two studies concerning EC knowledge and attitudes were conducted among adult populations particularly in the Western region of the world (USA, Puerto Rico, and other countries) [20, 21]. Both studies indicated a gap in EC knowledge especially pertaining to its regulation and constituents.

In the Middle Eastern region, where cultures and demographics vary from those of the Western world, the topic of ECs is still under-researched. Our study was conducted specifically in Lebanon since it is a unique developing Middle Eastern country with a distinctive social fabric comprised of a mixture of liberal and conservative ideologies. Therefore, this study’s findings are of significance at a regional and global level, given that Lebanon represents a confluence between the Eastern and the Western worlds. Given that EC use is a newly emerging habit in Lebanon, where tobacco use is prominent, it is important to investigate the current level of EC knowledge, the attitude towards ECs, and the interplay between these two factors and the demographical characteristics of the population, including smoking and lifestyle habits. Interestingly, none of the regionally or internationally conducted studies have examined the possible relationship between EC knowledge and the attitudes towards ECs. However, this correlation could potentially have crucial public health relevance and a bearing on the targets of EC policies and regulations.

Our extensive literature search also revealed that there are no available scales that can be adopted to solely assess the knowledge of EC and the attitude towards it. The two studies [20, 21] mentioned earlier did not include a validated scale for EC knowledge [20], nor did they employ an attitude scale [21] that is comprehensive enough to give an accurate assessment of attitude towards ECs. Hence, in this manuscript, we developed and validated an EC knowledge scale and employed it as an instrument to exclusively determine the participants’ level of knowledge on the topic. We have also developed an attitude scale that is exhaustive and useful in assessing the participants’ outlook towards ECs. Therefore, findings from this study will provide new insights into EC knowledge and attitude that have not been examined previously; it uses, for the first time, comprehensive scales that assess these two measures.

In this study, we aim to identify the factors that are associated with EC knowledge and attitude among Lebanese citizens, and to address the following objectives:

1. Assess and depict current demographical characteristics including smoking habits, in general, and EC use, in specific.
2. Determine the level of EC knowledge and the attitude towards ECs. This objective necessitates the development of a comprehensive attitude scale, and development and validation of a knowledge scale for ECs.
3. Determine the correlation between EC knowledge and the attitude towards ECs.
4. Identify the predictors of attitude towards ECs and determine which aspects of participants’ knowledge are associated with attitude.
5. Identify the predictors of EC knowledge, and determine which aspects of the participants’ attitude are associated with their knowledge of ECs.

## Methods

### Study design

We have carried out a cross-sectional study whereby a self-administered questionnaire was employed as an instrument for data collection to gather information pertaining to demographical and lifestyle characteristics, smoking habits in general and EC in particular, as well as knowledge and attitudes regarding the harms and proper use of ECs among Lebanese citizens.

### Sampling strategy

Our study population was comprised of a convenience sample of Lebanese pedestrians in diverse streets and districts in Beirut, the capital of Lebanon. These participants were approached randomly and asked if they were willing to fill in the questionnaire.

- Inclusion and exclusion criteria:
  Our target population consisted of adults between the ages of 18 and 64 (inclusive) with a Lebanese nationality. Non-Lebanese citizens, those younger than the age of 18 or older than 64, and those who can neither read nor understand English or Arabic were all excluded from our study.
- Sample size
  The target sample size has been calculated from a regression angle with low anticipated effect size of 0.1, a statistical power level of 0.8, 20 predictors and a probability level of 0.05 [22–24]. This specification resulted in a total sample size of 226. Accounting for missing data and non-response by inflating the sample size by 1.2 and by 1.25 respectively resulted in a sample size of 339 participants. Hence, we opted for a target sample size of 340 individuals randomly selected from Beirut. Three hundred ninety-two surveys were filled and assessed for eligibility; 40 surveys were discarded according to the aforementioned exclusion criteria, leaving our study with a total sample size of 352 completed questionnaires.

### Concepts and indicators

English and Arabic versions of the questionnaire were available to satisfy the participants’ language of preference. Two validation studies were conducted: one for the translation of the entire questionnaire to ensure reliability and consistency in the Arabic and English versions, and the second for the knowledge scale alone to validate its effectiveness in correctly reflecting the knowledge level of ECs among participants. The description of the knowledge scale and the results of the validation studies are included in section 6 (results section) and further explained in Additional file 1: Appendix B.

The questionnaire employed in this study consisted of 6 sections as detailed below:

#### Section 1

This section assessed the demographics of the sample population and ensured satisfaction of the inclusion criteria as per age and nationality. Questions included in this section were related to demographic and socioeconomic characteristics of the participants, such as age, sex, nationality, occupation, level of education, and monthly income.

#### Section 2

To assess smoking habits and the sample proportion of the different smoking types, participants were asked about their smoking behavior or the lack thereof. Smoking cessation was also studied by asking participants whether or not they have quit or considered quitting smoking, and if applicable, the type of smoking they gave up and their smoking cessation method.

#### Section 3

This section assessed the lifestyle habits of the participants including perceived regular exercise and healthy dieting. In addition, the participants were asked if they drink alcohol or coffee and whether regular or EC smoking coincides with alcohol or coffee drinking.

#### Section 4

This section explored EC habits. Participants were asked if they had heard of ECs and if applicable, from where. Participants were also asked whether they use ECs and their reasons for using or abstaining from it. EC users were asked about the flavor of their ECs, the presence of nicotine, the duration and frequency of their use, and the level of satisfaction gained by the use of ECs relative to regular tobacco, if they smoked both.

#### Section 5

This section evaluates the attitudes of the participants towards ECs using an attitude scale that incorporates questions inspired from various sources and modified to become applicable to our study [1, 8, 9, 21, 25, 26].

The questions reflecting a positive attitude when answered in the affirmative include those discussing EC use in places forbidding other types of smoking, its social acceptability, its effectiveness and acceptability as a smoking cessation tool, and its ability to replace tobacco cigarettes. Affirmations that it should be recommended to a nonsmoker and experimented with for pleasure also portray a positive attitude. The questions which reflect more of a negative attitude towards ECs when answered in the affirmative entail ECs’ governmental regulation, their harm, their reliance potential, and whether the participant would label an EC user a “smoker”. A score was computed by adding all answers that count towards a positive attitude. The higher the score, the more a participant holds a positive attitude. The approach used to compute and analyze the attitude related questions is described in the data analysis section of this manuscript.

In addition to the literature search that was used to develop this section of the questionnaire, the attitude scale was preliminarily assessed and reviewed by three experts in smoking cessation and modified and finalized based on their comments to ensure that it was comprehensive and detailed enough to capture the participants’ attitudes towards ECs from various perspectives.

It is important to highlight here, that attitudes are subjective and could neither be deemed as correct or incorrect nor be compared to a certain reference. For this reason, participants can be only described as holding either a more positive or a more negative attitude on an overall spectrum based on their raw attitude scores without claiming that the positive or negative attitudes they hold are correct or justifiable.

#### Section 6

To evaluate the participants’ knowledge of ECs, a knowledge scale was developed after a thorough literature search and consultation with three experts in the field of smoking and smoking cessation who assessed and ensured the comprehensiveness of the knowledge scale. Information pertaining to ECs, its utility, harms, and benefits was gathered from various sources, compiled, and converted into true and false questions ultimately producing a comprehensive scale [3, 11, 12, 21, 27–32]. More details on these questions, their correct answers, and the resources from which these questions were adopted are presented in Table 3 and Additional file 1: Appendix A.

A validation study was then performed on this scale to assess its accuracy in detecting the knowledge level of experts and non-experts in ECs. Experts in the field of smoking and smoking cessation were expected to achieve a high knowledge score compared to non-experts who were expected to achieve a low score. This was confirmed by statistical tests of significance. The 17 experts approached included doctors in pulmonary and family medicine and nurses who hold certificates in smoking cessation. For comparison, 28 non-experts were also approached. The experts and non-experts were not part of the research team in order to ensure objectivity and impartiality in their assessment and evaluation of the knowledge scale. The knowledge score was then computed by summing up all the correctly answered questions and a higher score reflected more knowledge about ECs. Details of the validation study for the accuracy of the EC knowledge scale in identifying individuals who are knowledgeable on the topic are briefly mentioned in the results section and are fully explained in Additional file 1: Appendix B.

### Data collection

Data was collected between December 2018 and March 2019, by randomly approaching people on various streets and districts of Beirut. These areas exhibit diversity in background and socio-economic status and provide a relatively representative sample of the Lebanese community in Beirut. Approached participants were informed of the objectives of the study and were notified that their participation is voluntary, confidential and anonymous, and that they reserve the right to withdraw at any point while filling the survey. The answers to knowledge questions were not revealed before or during the filling of the questionnaire to avoid introducing any form of bias to their responses. After the oral consent was obtained, and upon completion of the survey, pamphlets containing the answers to the knowledge section of the survey (i.e. the relevant facts regarding E-cigarettes) were given to the participants in order to raise awareness about ECs and correct any misconceptions on the topic.

### Plan of analysis

A value of “1” was assigned to each correct answer the participants gave upon filling the knowledge section of the questionnaire whereas a “0” was given to an incorrect answer. Similarly, for the attitude questions, a value of “1” was attributed to answers representing a positive attitude and “0” was allocated to those representing a negative attitude. The knowledge and attitude scores for each participant were obtained by summing up the values earned on all the answers pertaining to each section. The maximum scores attainable on the attitude and knowledge scales were 13 and 16 respectively.

Since scores on both scales were not normally distributed, a Spearman’s correlation was carried out to assess the crude association between knowledge of and attitude towards ECs.

The scores of the knowledge and attitude scales were then dichotomized to binary random variables by assuming a cutoff of 75%. In this regard, a participant who answered 75% or more of the questions correctly was considered knowledgeable in ECs, otherwise, he/she was considered not knowledgeable. Similarly, a participant with 75% or more answers denoting positive attitude was categorized as having good attitude, otherwise, the participant was classified as having a more negative attitude toward ECs. This percentage cutoff point was adopted by other studies assessing attitudes, knowledge, and awareness in other disciplines [33].

The attitude section contained 13 questions and thus a score of 9 and above implied a more positive attitudes towards ECs. The knowledge section of the questionnaire contained 16 questions and thus a score of 12 and above denoted a higher level of knowledge. Any score below 9/13 and 12/16 implied a more negative attitude towards ECs and less knowledge respectively. Consequently, we recoded scores above the cutoff into “1” and those below the cutoff into “0”. These two newly recoded dichotomized random variables were used as outcomes for attitudes and knowledge.

Simple and multiple logistic regressions were carried out to identify the covariates that are significantly associated with the dichotomized knowledge and attitude scores, and the corresponding crude and adjusted odds ratios (ORs), 95% confidence intervals (CI) and *p*-values were all generated. The results of the bivariate analyses showing the predictors of attitude and knowledge are summarized in Appendices C and D. Significant factors (*p* ≤ .05) and those with a p-value of .20 and below in simple logistic regression were considered eligible to be included in the multiple logistic regressions for both attitude and knowledge. Since the standard significance level of .05 was shown to fail in selecting important covariates known to be associated with the outcome [34–37], the cutoff for eligibility of inclusion in the multiple logistic regression was increased to a significance level of .2 (*p* ≤ .2). This ensured that covariates that could potentially be associated with the outcome were all considered in the multiple regression model.

The aim was to identify the predictors that maintained or gained a significant association with EC knowledge and attitude after adjusting for universal covariates such as age and sex, as well as other factors.

### Ethical considerations

Before conducting this study, an approval by the institutional review board at the American University of Beirut (IRB ID: SBS-2018-0608) was sought and attained. All ethical considerations were honored throughout the study period.

## Results

Our validation studies ensured the accuracy of the knowledge scale in correctly classifying participants as either knowledgeable in ECs or not. In addition, experts had significantly higher EC knowledge scores compared to non-experts (*p* < .001). To ensure accuracy of translation, 26 participants, who were not part of the study, were approached to each fill both English and Arabic versions of the questionnaire. Precision of the translation between the Arabic and English versions was confirmed using two statistics: percent agreement and Cohen’s kappa statistic. Our results showed that the percent agreement was high with an overall average of 95%. Similarly, Cohen’s kappa values were also high with an overall mean value of 0.9. The high values of the percent agreement and kappa statistics along with their statistical significance indicate consistency in the translation and reproducibility of the responses between the English and Arabic versions of the questionnaire. Full explanation of the validation studies is included in Additional file 1: Appendix B.

Our analysis was initiated by data cleaning whereby outliers and data entry errors were detected and rectified. Descriptive statistics, graphical and numerical, were carried out to determine the frequency distribution of each variable. All variables, except for age, were measured on a categorical scale. The mean age with its standard deviation (SD) and the count with valid percent are presented in the descriptive tables shown in the result section (Tables 1 and 2). Valid percentages were used in the calculations and characteristic distribution reports in Tables 1, 2, 3 and 4. Missing data was not an issue in this study because the maximum observed percentage of missing responses did not exceed 8% in any category or subcategory. It is important to note that in the reported Tables some counts do not add up to the total sample size of 352 (or corresponding subtotal), attributable to the fact that some questions are not applicable to the respondent. Observations with available non-missing values were included automatically in the analysis.

**Table 1:** Demographics, socioeconomic, smoking habits, and lifestyle characteristics distribution of our study participants (*N* = 352) in Beirut, Lebanon between December 2018 and March 2019

	No.	Valid %
Age (Mean ± SD)	30.29 ± 11.78	
Sex		
Male	198	56.6
Female	152	43.4
Level of education		
Bachelor’s degree	156	44.3
Graduate school	100	28.4
High school level or equivalent	52	14.8
Intermediate level or below	33	9.4
Technical /vocational study	7	2.0
No schooling completed	4	1.1
Is your occupation or major health related?		
Yes	228	78.1
No	64	21.9
Smoking Habits		
Do you currently smoke?		
Yes	162	46.2
No (non-smokers)	151	43.0
Quit/quitting smoking	38	10.8
If you smoke, what do you smoke? (Circle all that apply)		
Cigarettes	137	52.7
Hookah^a^	68	26.2
Cigars	13	5
E-cigarettes	30	11.5
Dokha^b^	6	2.3
Pipe^c^	1	0.4
Other	5	1.9
What form of smoking did you quit/are you quitting? (Circle all that apply)		
Cigarettes	44	58.7
Hooka	20	26.7
Cigars	4	5.3
Dokha	3	4
E-cigarettes	3	4
Pipe	0	0
Other	1	1.3
What methods of smoking cessation have you used/are you using? (Circle all that apply)		
Reducing number of cigarettes per day	39	42.8
E-cigarettes	12	13.2
Behavioral therapy	5	5.5
Nicotine patches	3	3.3
Medication	2	2.2
Nicotine gum	1	1.1
Other	29	31.9
Have you ever thought of quitting smoking?		
Yes	132	72.5
No	50	27.5
Lifestyle characteristics^a^		
Do you exercise regularly?		
No	197	56.0
Yes	155	44.0
Do you believe you follow a healthy diet?		
No	191	54.3
Yes	161	45.7
Do you drink alcohol?		
No	195	55.4
Yes	157	44.6
Do you drink coffee?		
Yes	281	80.5
No	68	19.5

^a^Hookah or waterpipe: “a smoking device that consists of a bowl mounted on a vessel of water which is provided with a long tube and arranged so that smoke is drawn through the water where it is cooled and up the tube to the mouth” [38]

^b^Dokha: is tobacco blended with “barks, herbs, spices, dried flowers or dried fruit” and smoked in a particular pipe called “midwakh”. It has a high nicotine content, around five times that of a normal cigarette [39]

^c^Pipe: “a device for smoking usually consisting of a tube having a bowl at one end and a mouthpiece at the other” [40]

^d^The study did not provide standard definitions for “exercise regularly”, “healthy diet”, “drink alcohol”, or “drink coffee”; the participants answered these questions based on their subjective interpretation of these terms

**Table 2:** Frequency distribution of E-cigarette smoking habits among study participants (N = 352) in Beirut, Lebanon between December 2018 and March 2019

E-cigarette Habit Question	No.	Valid %
Have you heard of E-cigarettes		
Yes	318	90.3
No	34	9.7
If yes, where have you heard of E-cigarettes? (Circle all that apply)		
Friends	232	34.0
Social media	172	25.2
Advertisements	117	17.2
Family	84	12.3
University/school	48	7.0
Doctor	20	2.9
Center for smoking cessation	9	1.3
Do you use E-cigarettes?		
No	295	89.1
Yes	36	10.9
What flavor of E-cigarettes do you use? (Circle all that apply)		
Fruit	27	45.8
Tobacco	10	16.9
Menthol/mint	9	15.3
Candy	7	11.9
Coffee	3	5.1
Other	3	5.1
#Does the E-cigarette you use contain nicotine?		
Yes	30	83.3
No	6	16.7
How long have you been using E-cigarettes (months)? (Mean)	11.99	
What made you start smoking E-cigarettes? (Circle all that apply)		
Taste	20	28.6
Social smoking	12	17.1
Quit smoking	11	15.7
Healthier	11	15.7
Trend	10	14.3
Effects (relaxant, etc. …)	3	4.3
Other	3	4.3
Why do you not use E-cigarettes? (Circle all that apply)		
I do not smoke	130	34.9
Never considered it	118	31.6
Harmful/unhealthy	73	19.6
Expensive	34	9.1
No access	18	4.8
Compared to regular cigarettes (or other tobacco products) how much satisfaction do you get from E-cigarettes?		
Less	19	57.6
Same	8	24.2
More	6	18.2

**Table 3:** Frequency distribution of E-cigarettes knowledge responses of participants who have heard of E-cigarettes (*N* = 318) in Beirut, Lebanon between December 2018 and March 2019

Knowledge question	No.	Valid %
E-cigarettes are associated with bladder cancer		
False - Wrong Answer	251	75.4
True - Correct Answer	82	24.6
E-cigarettes are FDA Approved		
True - Wrong Answer	247	74.4
False - Correct Answer	85	25.6
Some flavors of E-cigarettes are more harmful than others		
False – Wrong answer	232	70.1
True – Correct answer	99	29.9
Swallowing the liquid in E-cigarettes accidentally can cause poisoning that is potentially fatal		
False - Wrong Answer	183	55.3
True - Correct Answer	148	44.7
E-cigarettes are not associated with lung cancer		
True - Wrong Answer	181	54.4
False - Correct Answer	152	45.6
E-cigarettes impair lung and heart functions		
False – Wrong answer	110	50.9
True – Correct answer	216	49.1
E-cigarettes do not contribute to second hand smoking		
True - Wrong Answer	153	46.2
False - Correct Answer	178	53.8
E-cigarettes can have an effect on fetal development		
False - Wrong Answer	132	39.6
True - Correct Answer	201	60.4
Nicotine is present in most E-cigarettes		
False - Wrong Answer	129	38.9
True - Correct Answer	203	61.1
Harmful flavorings and toxins are found in the E-cigarette aerosol		
False - Wrong Answer	125	38.0
True - Correct Answer	204	62.0
E-cigarettes are harmless		
True - Wrong Answer	114	34.7
False - Correct Answer	215	65.3
Some components of the liquid found in E-cigarettes can cause harmful lung conditions		
False - Wrong Answer	111	33.5
True - Correct Answer	220	66.5
E-cigarettes are not addictive		
True - Wrong Answer	110	33.0
False - Correct Answer	223	67.0
E-cigarettes are suitable for pregnant women		
True - Wrong Answer	35	10.5
False - Correct Answer	298	89.5
E-cigarettes are suitable for children		
True - Wrong Answer	22	6.6
False - Correct Answer	310	93.4

**Table 4:** Frequency distribution of attitude towards E-cigarettes responses of participants who have heard of E-cigarettes (*N* = 318) in Beirut, Lebanon between December 2018 and March 2019

Attitude question	No.	Valid %
Should E-cigarettes be recommended to a nonsmoker?		
No - Negative Attitude	298	90.3
Yes - Positive Attitude	32	9.7
Do you think E-cigarettes are harmful for health?		
Yes - Negative Attitude	272	84.2
No - Positive Attitude	51	15.8
Should the use of E-cigarettes be allowed in places that do not allow smoking?		
No - Negative Attitude	259	79.7
Yes - Positive Attitude	66	20.3
Would you consider someone who uses E-cigarettes a smoker?		
Yes - Negative Attitude	252	77.1
No - Positive Attitude	75	22.9
Do you think the use of E-cigarettes can lead to reliance?		
Yes - Negative Attitude	243	76.4
No - Positive Attitude	75	23.6
Do you think the government should regulate the use of E-cigarettes?		
Yes - Negative Attitude	226	70.0
No - Positive Attitude	97	30.0
Do you feel more comfortable using or openly talking about smoking E-cigarettes, compared to cigarettes?		
No - Negative Attitude	217	69.1
Yes- Positive Attitude	97	30.9
Do you feel it is more socially acceptable to smoke E-cigarettes, compared to cigarettes?		
No - Negative Attitude	183	56.7
Yes- Positive Attitude	140	43.3
Should E-cigarettes be used as a replacement for regular cigarettes?		
No - Negative Attitude	180	55.7
Yes - Positive Attitude	143	44.3
Do you think it is acceptable to experiment with E-cigarettes for pleasure?		
No - Negative Attitude	182	55.7
Yes - Positive Attitude	145	44.3
Do you think using E-cigarettes would be an effective way to help in smoking cessation?		
No - Negative Attitude	165	51.4
Yes - Positive Attitude	156	48.6
Do you think it is acceptable to use E-cigarettes as a smoking cessation method?		
No - Negative Attitude	159	49.2
Yes - Positive Attitude	164	50.8
Do you think E-cigarettes can help people cut down on cigarettes or quit smoking?		
No - Negative Attitude	147	46.1
Yes - Positive Attitude	172	53.9

### Objective 1: assess and depict current demographical characteristics alongside habits of smoking in general and EC use in specific

#### Demographics

Results pertaining to the demographical distribution of our study population are displayed in Table 1. Our sample was comprised of 352 participants of which 56.6% were males and 43.4% were females. Our participants had a mean age of 30.3 ± 11.8 SD, median age of 26. 44.3% of them were holders of a bachelor’s degree as their highest degree, and 98.8% were literate. Our calculated literacy value included participants who attended some schooling. Our sample sociodemographic measurements showed a minimal margin of difference with respect to those of the Lebanese population in 2019 with a sex distribution of 50.3% males and 49.7% females, a median age of 29.6 years, and a literacy value of 91.4% [41–44] .

Among EC users (*N* = 36), 58.3% were male, 1.7% were female, 78.8% did not work in a health-related occupation or enroll in health-related majors, and 86.3% attained a bachelor’s degree or higher.

#### Smoking habits and lifestyle characteristics

**Fig. 1:**
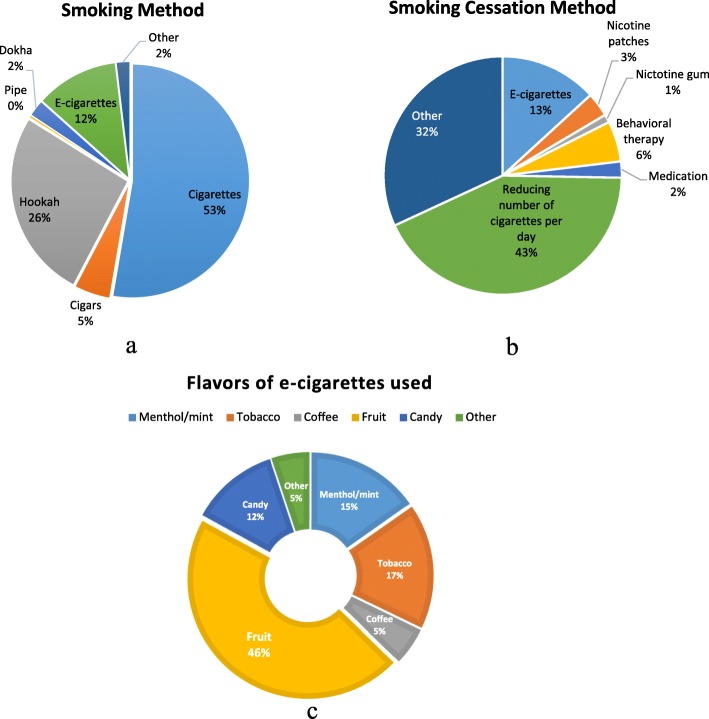
Frequency distribution of: (**a**) Types of smoking (Hookah or waterpipe: “a smoking device that consists of a bowl mounted on a vessel of water which is provided with a long tube and arranged so that smoke is drawn through the water where it is cooled and up the tube to the mouth” [38]. Dokha: is tobacco blended with “barks, herbs, spices, dried flowers or dried fruit” and smoked in a particular pipe called “midwakh”. It has a high nicotine content, around five times that of a normal cigarette [39]. Pipe: “a device for smoking usually consisting of a tube having a bowl at one end and a mouthpiece at the other” [39]) used by the participants with *N* = 260 total count for this subcategory, (**b**) Smoking cessation methods among participants who have quit or are in the process of quitting smoking with *N* = 91 total count for this subcategory, (**c**) E-cigarette flavors used by the E-cigarette smokers among participants with *N* = 59 total count for this subcategory

Out of those that filled the survey, 46.2% were smokers, 43.0% were non-smokers and 10.8% quit or were in the process of quitting smoking. Cigarettes were the most common means of smoking (52.7%) followed by hookah (also known as argileh) (26.2%) and EC (11.5%) (Fig. 1a). Of the participants who quit or were in the process of quitting smoking, the majority attempted cessation of cigarettes and hookah (58.7 and 26.7% respectively), mostly by reducing the number of cigarettes consumed per day (42.8%) over other cessation methods like EC, nicotine patches and gum, behavioral therapy or medication (Fig. 1b and Table 1). Hence, the majority of the participants did not resort to ECs to aid with their smoking cessation efforts.

The general lifestyle characteristics of our study population are also displayed in Table 1. Of the participants, 56% do not believe they exercise regularly, 54.3% believe they do not follow a healthy diet, and 44.6 and 80.5% are alcohol and coffee consumers respectively.

A bivariate analysis was conducted to assess a possible association between drinking coffee or alcohol and the participants’ habits in smoking either regular cigarettes or ECs. However, neither coffee nor alcohol drinking habits were significant predictors of smoking regular cigarettes. Drinking alcohol was indeed a significant predictor of EC smoking whereby participants who consumed alcohol were three times more likely to take up EC smoking (unadjusted OR = 3.006, 95% CI = [1.426, 6.337], *p* = .004). However, drinking coffee was not a significant predictor of the EC smoking habit.

#### E-cigarette habits

88.7% of the study population has heard of ECs mostly through friends, advertisements, social media, and family (34, 25.2, 17.2%, 12.3 respectively) (Table 2). Thirty-six out of the 352 participants (10.2%) were EC users. The flavors most commonly used were fruit (45.8%), tobacco (16.9%), and menthol/mint (15.3%) (Fig. 1c). Moreover, 83.3% of the aforementioned participants used nicotine-containing ECs (Table 2). While most of the EC smoking participants smoked EC on a daily basis (44.7%), a significant amount (36.8%) claimed to use them solely for social smoking purposes.

Among EC users, most participants initiated its use for its taste (28.6%), followed by those who took it up for social smoking reasons (17.1%), to quit smoking (15.7%), to use it as a healthier alternative to regular tobacco smoking (15.7%), and to follow the trend (14.3%). Only 18.2% of these participants reported getting more satisfaction from ECs compared to regular cigarettes or other tobacco products (Table 2).

A discrepancy was noted between the number of EC users that were reported in Table 1 (where a frequency of EC users of 30 is shown) and the number reported in Table 2 (where a frequency of EC users of 36 is shown). This inconsistency in the number of EC users could be due to errors in answering the question “If you smoke, what do you smoke?” which permitted more than one answer to be selected by the respondent and resulted in the frequency of 30 EC smokers. However, it is important to note that when analysis was carried out using either value, the estimates of the parameters and inferences were comparable. Because the value of 36 was obtained from a more direct question concerning the participants’ EC use (“Do you use E-cigarettes?” Yes/No), we concluded that it is the more reliable result and included this value in our analysis.

### Objective 2: determine the level of knowledge and attitude towards ECs

#### Level of knowledge of the participants towards EC

The mean knowledge score was 8.93 ± 4.12 SD (median Me = 9.00, lower quartile Q1 = 6.00, upper quartile Q3 = 12.00, interquartile range IQR = 6.00) with observed scores ranging from zero to 16 (16 representing the maximum level of knowledge). The mean of 8.93 is lower than the cutoff of 12 that was designated for a high level of knowledge. Specifically, 63.3% of our study population exhibited a lower level of EC knowledge with scores falling below this cutoff point.

**Fig. 2:**
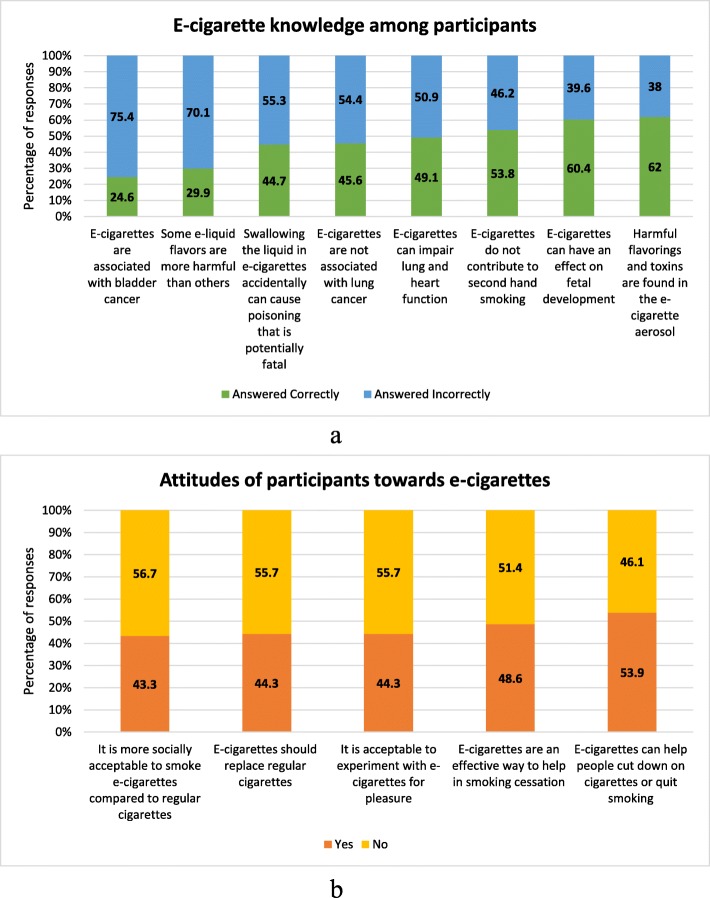
Frequency distribution of questions on EC-cigarettes that participants had: (**a**) the least knowledge of, and (**b**) the most negative attitude towards

The participants’ gap in EC knowledge was made apparent by several questions that were commonly answered incorrectly (Fig. 2a). 46.2% of the respondents falsely believed that ECs do not contribute to second hand smoking, 70.1% of the participants had the wrong perception that flavorings do not differ in their extent of harm. More than half of all respondents showed a lack of knowledge concerning ECs’ association with lung cancer, bladder cancer, and an impaired lung and heart function (54.4, 75.4, and 50.9% respectively). The questions in the knowledge scale and the distributions of the answers are displayed in Table 3.

#### Level of attitude of the participants towards EC

The participants had a mean attitude score of 4.23 ± 2.95 SD (Me = 4.00, Q1 = 2.00, Q3 = 6.00, IQR = 4.00) with scores ranging from zero to 13 (13 representing the maximum score and the most positive attitude). The mean of 4.23 is lower than the positive attitude cutoff point of 9. As depicted in Fig. 2b, a relatively good percentage of participants exhibited a positive attitude towards some aspects of ECs.

Our results, showed that 43.3% of the respondents felt it was more socially acceptable to smoke ECs compared to regular cigarettes, and a good proportion of 44.3% deemed it acceptable to experiment with ECs for pleasure. 48.6% thought that ECs were effective as a smoking cessation method, 53.9% believed that ECs could help people cut down or quit smoking, and 44.3% believed that ECs should replace regular cigarettes. The distribution of answers to all the questions included in the attitude scale are displayed in Table 4.

### Objective 3: correlation between attitude towards ECs and EC knowledge

The EC attitude score was significantly inversely correlated with EC knowledge score with *r* = −.30 (Spearman’s rho since the two variables are not normally distributed), and *p* < .0001. This indicates that as EC knowledge increases, the positive attitude towards it decreases and the inverse is also true.

### Objective 4: predictors of attitude towards ECs

Using the attitude score as a dichotomized binary outcome, simple logistic regressions were performed and the corresponding results were displayed in Additional file 1: Appendix C. Multiple logistic regressions were also carried out and the final multivariable analysis model for positive attitude was displayed in Table 5. Our multiple logistic regression model was adjusted for age and sex (universal covariates) and other eligible variables pertaining to EC knowledge (unadjusted *p* ≤ .20). Including knowledge covariates into the multivariable model is important to identify the EC knowledge variables that were significantly associated with the attitude towards ECs.

**Table 5:** Multiple logistic regression of the outcome attitude towards E-cigarettes showing adjusted associations with its covariates^**₮**^

Covariate		No. (%)	Adjusted Odds Ratio	95% Confidence Interval for Odds Ratio		*P*-value
				Lower Limit	Upper Limit	
E-cigarettes are addictive		223 (67.0)	.231	.080	.663	.006*
Use E-cigarettes		36 (10.9)	6.257	1.604	24.404	.008*
Sex	Male (reference)	198 (56.6)	.225	.072	.704	.010*
	Female	152 (43.4)				
E-cigarettes are harmful		215 (65.3)	.245	.080	.756	.014*
E-cigarettes impair lung and heart function		216 (66.3)	.301	.092	.984	.047*

* *p* ≤ .050 **₮** multivariable analysis was adjusted for age and adverse effects of nicotine smoking (effects on children, fetus, pregnant women, and risk of bladder cancer)

Our multivariable analysis identified several factors that exhibited significant associations with attitude towards EC. Our results showed that participants who consumed ECs were six times more likely to have a positive attitude (OR = 6.257, 95% CI = [1.604, 24.404.], *p* = .008) compared to non-EC users, whereas females were four times less likely to harbor a positive attitude compared to males (OR = .225, 95% CI = [.072, .704], *p* = .010).

As for the elements of knowledge that were associated with attitude, our results showed that individuals who were aware that ECs are addictive and harmful were almost four times less likely to have a positive attitude towards ECs (OR = .231, 95% CI = [.080, .663], *p* = .006 and OR = .245, 95% CI = [.080, .756], *p* = .014 respectively) (Table 5). In addition, participants who recognized that ECs impair lung and heart function were three times less likely to have a positive attitude (OR = .301, 95% CI = [.092, .984], *p* = .047). Hence, these results suggest that being knowledgeable of these harmful effects correlated with a more negative attitude towards ECs. This finding is supported by the inverse association between higher knowledge and positive attitude that was detected in the Spearman’s correlation.

All the other covariates that were individually significant predictors for attitude (*p* ≤ .05) in the bivariate analysis (Additional file 1: Appendix C) were no longer statistically significant when combined with the aforementioned predictors in the multivariable analysis (Table 5). Similarly, the factors that were not significant but eligible to enter the multivariable analysis (*p* ≤ .20) did not display a significant association with attitude in the multivariable analysis.

### Objective 5: predictors of knowledge of EC

Using the dichotomized binary knowledge score as an outcome, simple logistic regressions were carried out and the corresponding results were displayed in Additional file 1: Appendix D. Multiple logistic regressions were also conducted and the final multivariable analysis model for higher level of knowledge was displayed in Table 6. After adjusting the multiple logistic regression model for age and sex (both universal covariates), we identified several factors that exhibited significant associations with EC knowledge including EC use, healthy diet and regular exercise, health-related occupation and some factors related to attitude. In specific, covariates that showed an inverse association with knowledge were EC use and the participants’ beliefs that they follow a healthy diet (Table 6). EC users were found to be three times less likely to have attained a higher level of knowledge (OR = .304, 95% CI = [.107, .865], *p* = .026), and participants who believed that they follow a healthy diet were about half as likely to have a high level of knowledge (OR = .511, 95% CI = [.272, .962], p = .026). The covariates that exhibited a positive significant association with knowledge were health-related occupation/major and the participants’ belief that they exercise regularly. In this respect, participants who work in a health-related occupation or are enrolled in a health-related major as well as those who believe they exercise regularly were twice as likely to have higher EC knowledge (OR = 2.408, 95% CI = [1.233, 4.705], *p* = .010 and OR = 2.173, 95% CI = [1.158, 4.077], *p* = .016 respectively).

**Table 6:** Multiple logistic regression of the outcome knowledge of E-cigarettes showing adjusted associations with its covariates ^Ŧ^

Covariate	No. (%)	Adjusted Odds Ratio	95% Confidence Interval for Odds Ratio		*P*-value
			Lower Limit	Upper Limit	
Think E-cigarettes are not harmful for health	51 (15.8)	.122	.035	.434	.001*
Occupation or major is health related	64 (21.9)	2.408	1.233	4.705	.010*
Believe to exercise regularly	155 (44.0)	2.173	1.158	4.077	.016*
Think E-cigarettes can help people cut down on cigarettes or quit smoking	172 (53.9)	.501	.285	.882	.017*
Use E-cigarettes	36 (10.9)	.304	.107	.865	.026*
Believe to follow a healthy diet	161 (45.7)	.511	.272	.962	.026*

* *p* ≤ .050 ^**Ŧ**^ multivariable analysis was adjusted for age and sex

Aspects of the attitude scale that were associated with less knowledge included the positive perception that ECs can help people cut down on cigarette consumption or quit smoking, whereby participants who harbored this positive attitude were half as likely to have high knowledge (OR = .501, 95% CI = [.285, .882], *p* = .017). Moreover, participants who positively perceived that ECs are not harmful for health were eight times less likely to have a high level of knowledge (OR = .122, 95% CI = [.035, .434], *p* = .001). Hence, these results suggest that participants with the previously specified positive attitude towards ECs were less likely to have high levels of knowledge and be classified as knowledgeable in ECs. This finding is supported by the inverse correlation detected between knowledge and attitude towards ECs.

All other covariates in the bivariate analysis that were individually significant predictors of a high level of knowledge (*p* ≤ .05) (Additional file 1: Appendix D), as well as the factors that were eligible to enter the multivariable analysis (*p* ≤ .20) were no longer statistically significant when entered in the multivariable analysis (Table 6).

## Discussion

We propose a novel study identifying factors associated with the knowledge and attitude towards ECs among Lebanese participants. This study explored the nature of the correlation between attitude and knowledge and determined the specific aspects of knowledge that affect the participants’ attitudes towards ECs as well as aspects of attitudes that affect their knowledge. A thorough literature search revealed that there are no comprehensive knowledge or attitude scales available and that can be employed in the context of a Middle Eastern developing country like Lebanon. Hence, in order to achieve our objectives, we developed and validated our own scales. The novelty of this study is twofold: 1- filling a regional gap in the literature about this specific EC topic; 2- proposing an EC attitude scale and a comprehensive and useful knowledge scale.

Our study results revealed that there is an inverse significant correlation between knowledge of and attitude towards ECs, indicating that a higher level of knowledge is associated with a more negative attitude. Those who are more knowledgeable about EC use would also know more about its potential harms and therefore, would perceive it more negatively than those who lack knowledge about it.

Our study also showed that people started using ECs mostly because of its flavor. According to our data, the most popular flavors for the EL were fruit, tobacco, and menthol/mint, similar to the findings drawn by a previous study [25]. One possible explanation is that these flavors resemble those found in the more commonly used cigarettes and hookah making them more familiar to EC users. The hookah is popular in our culture showing the second highest frequency of use in our study (26.2%), following cigarettes (52.7%). The hookah is enjoyed because of its variety of available flavors. ECs have the potential to be used as a portable hookah which might justify our study’s conclusion that the most common reason to start EC smoking was its taste. Compared to Middle Eastern countries, in the Western region the hookah is less popular and less culturally tied. Therefore, studies conducted in Western countries focused more on health-related reasons of EC use [45]. In this regard, the EC use among participants was attributed to its perceived reduced harmful effects compared to tobacco, rather than to its flavors [45]. Given that the EC flavors render it as an auxiliary to hookah, many users of ECs compare the taste between these two smoking devices; therefore, they could uphold a negative attitude towards ECs if they do not get the same level of nicotine satisfaction or if its taste does not live up to that of hookah.

Most of the EC users that participated in our study were of a young age (mean age 25.8); a factor substantiated by Goniewicz et al.’s claim that “EC users tend to be younger” [25]. Since ECs are relatively new devices, a young age group would like to follow such emerging trends. Moreover, the majority of the participants in our study heard about ECs through their friends and family (46.3%), followed by social media (25.2%) and advertisements (17.2%). This could be attributed to peer pressure and the influence of friends, both of which are more common among the young population, the predominant age group of our study, particularly regarding popular trends. Our findings were in line with those of a study conducted on EC users in Atlanta, Georgia where most participants reported hearing about ECs through friends and family and the minority through TV news stories and advertisements [8].

Concerning the attitudes of the participants towards ECs, our data was not congruent with other studies in the literature. While 88.9% of participants thought that EC use should be allowed in places that do not allow regular smoking [8], the data in our study showed only 20.3% of such a positive attitude towards ECs. Moreover, while 80.6% thought that ECs help in quitting smoking [8], a comparative question in our study addressing its use as an effective smoking cessation method obtained a 48.6% positive response. Furthermore, there was a 27.8% belief that ECs should be FDA regulated [8], whereas our study showed a 70% belief that it should be governmentally regulated.

E-cigarettes entered the Lebanese market almost 4 years ago while it has been in the US market since the mid-2000s and its sales rose rapidly in 2007 [31, 46]. Additionally, in Lebanon, EC import and sale was regulated in 2013, even before its introduction to the market because it was deemed comparable in harm to cigarettes by the Ministry of Health [46]. On the other hand, in the US, the FDA officially regulated ECs in 2016, around 10 years after their introduction into the market and its widespread use [31]. Hence, relative to the US population, the Lebanese population did not have enough exposure to enrich their knowledge in ECs and their attitudes could have been influenced by the government’s initial negative perception towards this smoking device. Furthermore, the recent anti-tobacco law enforced in 2012 in Lebanon could have contributed to an overall negative outlook on smoking in general [46]. Therefore, the differences between our study and that conducted in Georgia [8] could be due to three reasons: 1- EC’s relatively new introduction to the Lebanese market and therefore, participants not knowing much about it and attributing their perception of tobacco smoking to EC smoking; 2- ECs being less socially acceptable in Lebanon compared to its tobacco counterparts which have existed for longer; 3- the recent implementation of the indoor smoking ban in Lebanon and consequently peoples’ increased concern with smoking in general.

Our study participants lack knowledge regarding the harms of ECs where more than half of the participants incorrectly answered questions about ECs’ association with lung cancer, bladder cancer, and the impairment of lung and heart function. In addition, around 70% did not know that some flavors were more harmful than others. A substantial percentage were unaware that ECs contribute to second hand smoking (46.2%), that they have an effect on fetal development (38.9%), and that most of them contain nicotine (38.9%). Our study is the first to develop a knowledge score, therefore, our results could not be compared.

Our study produced two multivariable models, one for a higher level of knowledge and another for a more positive attitude; these models accounted for confounders as well as other predictors including those related to demographics. In the model for higher knowledge, two variables behaved as positive predictors. First, those who admitted to a health-related major or occupation displayed a higher level of knowledge concerning ECs as their major and occupation must expose them to such knowledge related to ECs or equip them with the capacity to make better informed judgments about health. Second, participants who believed they exercised regularly also portrayed more knowledge which can be explained by their healthier lifestyle and thus their greater awareness of habits that could be harmful to one’s health. On the other hand, we obtained four negative predictors for higher knowledge. Participants who used ECs proved to have less knowledge on the topic, a factor that could possibly contribute to their choice of using this device. Those who believed they followed a healthy diet also had less knowledge as their perceived healthier lifestyle could have made them disinterested with ECs and therefore less likely to learn more about them. The perception that ECs are not harmful for health and that they are effective for smoking cessation are both positive attitudes towards ECs and negative predictors for knowledge. This relationship is substantiated by the inverse correlation between knowledge and attitude, where those who know more about ECs and its harms are more likely to view it negatively.

Concerning the attitude model, there was one positive and four negative predictors for a more positive attitude. Those who used ECs possessed a more positive attitude as a way to justify their use of the device. The knowledge that most ECs are addictive, harmful, and impair lung and heart function were all negative predictors of a positive attitude reiterated by the inverse correlation between knowledge of ECs and the attitude towards them.

In summary, there is a clear EC knowledge gap among the participants especially pertaining to certain areas like EL constituents’ hazards and ECs’ harmful effects on organ functions. In addition, there seems to be a generally negative attitude towards ECs among the respondents notwithstanding a more positive one towards its use for smoking cessation and experimentation for pleasure. Our study showed an inverse correlation between EC knowledge and attitudes towards ECs. Predictors for both knowledge and attitude were identified. Factors associated with correct knowledge of ECs include regular exercise and health-related occupations/majors. Meanwhile, EC use, thinking ECs are not harmful for health and that they could help in quitting smoking, and following a healthy diet were associated with incorrect EC knowledge. Predictors for positive attitude towards ECs included EC use and male sex; whereas, the knowledge that most ECs are addictive, impair heart and lung function, and are harmful were associated with a more negative attitude.

### Strengths and limitations of the present study

Efforts have been made to come up with scales that reflect the attitudes of participants towards EC [21], as well as their knowledge of specific aspects of this novel smoking tool such as its constituents and regulation [20]. These studies were limited in the variables they addressed within EC knowledge and attitude. Moreover, no studies were conducted thus far to assess how knowledge of and attitudes towards ECs are associated, and no scales were available to determine in a comprehensive manner the knowledge of EC and attitude towards it. Therefore, this study is novel because it: 1- contains an extensive EC knowledge scale as well as an equally exhaustive attitude scale and 2- examines how EC knowledge and attitude are interrelated, and 3- identifies factors that are associated with these two measures. This study is significant not only in filling a knowledge gap in this research area in Lebanon and the region, but also in generating the first useful knowledge and attitude scales of ECs that are comprehensive and that can be adopted regionally and internationally to specifically assess the level of knowledge pertaining to EC and attitude towards it.

We acknowledge that our study population was skewed towards a younger and more educated population; however, given that these two factors (age and education) did not show any significant association with neither the knowledge nor the attitude scores obtained, this skewness should not affect the generalizability of our results. Nevertheless, despite our attempts to choose districts representative of different demographics and socioeconomic status, including participants solely from Beirut poses some inevitable limitations on the generalizability and extrapolation of the results to other urban and rural areas of Lebanon.

Using a convenience sample also limits the generalizability of this study. To address this limitation, we made sure to maximize the number and variability of the sampled areas in Beirut. We have also ensured that the pedestrians were approached randomly with no criteria to our choice of responders other than the exclusion criteria, which we confirmed after approaching a prospective participant. This left minimal room for selection bias. Notwithstanding the effect that the convenience sample may have had on the external validity of the study, our sample population has shown to be comparable to the Lebanese population as there was a small margin of difference with regards to sex distribution, median age, and literacy [41–44].

We did not have an objective measure for quitting smoking, rather the participant answered subjectively. Without a standard definition for this measure (e.g. smoked 100 cigarettes in a lifetime and currently do not smoke), participants who quit smoking were grouped together regardless of how recently they quit or the frequency of their smoking habits prior to quitting [47]. Therefore, the value of 10.8% of individuals who quit or are in the process of quitting smoking could be an overestimate. Nevertheless, this value was used to observe the overall pattern of smoking in our sample and does not affect the results of our EC knowledge and attitude analysis.

## Public health significance, implications, and future direction

ECs are relatively new devices; therefore, people are not well-informed about their harms and benefits as was evidenced by the results of our study. For instance, participants scored lower on harm-related questions such as the effects of ECs on the heart, lungs, and bladder. They were also less aware that ECs contribute to second-hand smoking. Therefore, it is necessary to educate the population regarding ECs, specifically related to their harms. Studies such as ours are imperative in providing data useful in guiding the initiation of effective corrective measures regarding EC misconceptions. Reformatory actions should be undertaken through organization of awareness campaigns, drafting of policy briefs, and institutionalization of new and more stringent laws that regulate this new smoking device.

Although restricting our study to Beirut limits its generalizability, its results lay the foundation for future studies which could be carried out at a national level under the support of the Ministry of Health and other governmental agencies. This would garner a more holistic and profound understanding of the awareness and perception of the Lebanese community towards ECs and smoking in general. Considering that the level of EC knowledge would be predictably lower had this study been conducted in rural areas rather than in the capital and largest city of Lebanon, efforts to increase awareness should take place at a national level to capture the diverse demographical characteristics of the Lebanese community.

Awareness campaigns about ECs should target people of different age groups, educational levels, and socioeconomic status. Educating the population about ECs allows individuals to make more informed decisions about its consumption. Our results singled out specific aspects of misconceptions about ECs that could guide the mission and objectives of national awareness campaigns [48]. Attempts should be focused on the young in order to inform them about the harmful and addictive adverse effects of ECs when misused as devices for recreational purposes. This can be achieved by incorporating these awareness campaigns into school and university curricula as a mandated and integral component of general health education courses [49, 50].

Recommendations for the aforementioned initiatives were inspired from the outcomes of previous studies. In this regard, smoking cessation interventions implemented in classrooms were shown to be effective in reducing the prevalence of smoking among adolescents and increasing the propensity for smoking cessation in the US and Australia [49, 50]. Additionally, mass media smoking cessation campaigns ran at a national level in the US succeeded in increasing the awareness regarding the harms of smoking and the inclination for quitting this habit [48]. Therefore, such active measures should be taken in order to enlighten the population about the adverse effects of EC use and correct any misconceptions about the safety of its consumption.

Health care providers should also play a role in spreading awareness in the community by educating their patients on the attributable risks of ECs on health. Hence, understanding the attitude towards ECs and recognizing its misconceptions highlight the issues health care providers should address when informing patients about the use of ECs recreationally or for the purpose of smoking cessation.

More awareness could lead to a more negative attitude towards ECs, supported by the inverse association between knowledge and attitude. This is crucial because individuals displaying a positive attitude towards ECs could become more easily inclined to take up this smoking habit. Despite the small percentage of EC users in our study (11%), EC use was found to be significantly associated with a more positive attitude and less knowledge about ECs. This again suggests that individuals with a positive attitude towards ECs tend to be less knowledgeable and more enticed to use it as a smoking device.

## Conclusions

In light of the results of this study, actions to improve the level of knowledge are necessary to shape the attitude towards ECs and mitigate their use, especially for recreational purposes. Moreover, the government should take proactive measures to attenuate the spread of unjustifiable EC use in order to mitigate the burden of EC related cancer and pulmonary and cardiovascular diseases. A lack of serious intervention from governmental agencies and relevant ministries, and absence of laws such as the one that was recently introduced by the Lebanese government banning smoking in enclosed public areas [46], will allow the misconceptions surrounding ECs to propagate in the community leading to an exacerbation in disease burden and health care expenditure.

## Supplementary information

**Additional file 1 Appendix A** Knowledge scale of the questionnaire and corresponding answers. The knowledge section of the questionnaire used in this study asked the participants if certain statements pertaining to the general use of ECs are true or false. The correct answers to the questions are discussed in detail in Appendix A. **Appendix B.** Validation studies for the questionnaire: one for the translation from English to Arabic and one for the knowledge scale. A validation study that assessed the internal consistency between the answers of the English and Arabic versions of the questionnaire was carried out to determine the translation accuracy. Moreover, another validation study was carried out to assess the knowledge scale’s effectiveness in classifying individuals as knowledgeable or not in ECs. Detailed description of these validation studies and their corresponding results are included in Appendix B. **Appendix C.** Bivariate analysis of the outcome attitude towards E-cigarettes with its covariates. The unadjusted odds ratio and corresponding confidence interval, along with the P-value showing the unadjusted association between each covariate and the outcome “attitude” are tabulated and presented in Appendix C. **Appendix D.** Bivariate analysis of the outcome knowledge of E-cigarettes with its covariates. The unadjusted odds ratios and their corresponding confidence intervals, along with the *P*-values showing the unadjusted associations between the covariates and the outcome “knowledge” are tabulated and presented in Appendix D.

## Data Availability

The questionnaire and datasets used and/or analyzed during the current study are available from the corresponding authors upon reasonable request.

## Abbreviations

CI: Confidence interval
EC: Electronic cigarette
EL: E-liquid
FDA: The food and drug administration
IQR: Interquartile range
Me: Median
OR: Odds ratio
Q1: Lower quartile
Q3: Upper quartile
SD: Standard deviation
