# Supplementary material for: Knowledge and attitudes towards E-cigarette use in Lebanon and their associated factors
Source: BMC Public Health. 2020 Feb 28;20:278. doi: 10.1186/s12889-020-8381-x (PMC7049178; doi:10.1186/s12889-020-8381-x)
Supplement: Supplementary file 1 — Additional file 1 Appendix A Knowledge scale of the questionnaire and corresponding answers. The knowledge section of the questionnaire used in this study asked the participants if certain statements pertaining to the general use of ECs are true or false. The correct answers to the questions are discussed in detail in Appendix A. Appendix B. Validation studies for the questionnaire: one for the translation from English to Arabic and one for the knowledge scale. A validation study that assessed the internal consistency between the answers of the English and Arabic versions of the questionnaire was carried out to determine the translation accuracy. Moreover, another validation study was carried out to assess the knowledge scale’s effectiveness in classifying individuals as knowledgeable or not in ECs. Detailed description of these validation studies and their corresponding results are included in Appendix B. Appendix C. Bivariate analysis of the outcome attitude towards E-cigarettes with its covariates. The unadjusted odds ratio and corresponding confidence interval, along with the P-value showing the unadjusted association between each covariate and the outcome “attitude” are tabulated and presented in Appendix C. Appendix D. Bivariate analysis of the outcome knowledge of E-cigarettes with its covariates. The unadjusted odds ratios and their corresponding confidence intervals, along with the P-values showing the unadjusted associations between the covariates and the outcome “knowledge” are tabulated and presented in Appendix D. [file 12889_2020_8381_MOESM1_ESM.docx]

**Appendix A: Knowledge scale of the questionnaire and corresponding answers.**

The knowledge part of the questionnaire used in this study involves asking the participants if certain statements pertaining to the general use of ECs are true or false. The following are the correct answers to the questions discussed in details:

At the time when this study was conducted, ECs were still not approved by the US Food and Drug Administration (FDA) as a tool for smoking cessation. Harmful effects on the lungs in first-time smokers of ECs were noted, which explains why ECs should not be used by non-smokers (1). ECs do contribute to second hand smoking (2). In addition, not only is the nicotine in ECs not safe for use by children, but so is the toxic EL that comes in all ECs that is potentially fatal if swallowed by accident (3). In fact, studies have shown that swallowing EL can be fatal in both adults and children with the lethal doses of nicotine being between 0.5 and 1 mg/kg, and 0.1 mg/kg of their body weights respectively (3). ECs have been shown to impair lung function by reducing the ability of its cells to repair and by morphologically inducing COPD-like changes at the level of the lungs (4). Additionally, signs of lung cancer appeared in mice exposed to ECs (5), and bladder cancer specific carcinogens were found in the urine of EC users (6). Furthermore, EC smoking has been associated with damage to the heart. Specifically, the regular use of ECs induced a high blood pressure and amplified the risk of cardiac arrhythmias (7). ECs can be as addictive as smoked tobacco mainly due to nicotine being an active ingredient in various EC brands, which induces changes in the brain (US Department of Health and Human Services, 2016). There has been a rise in the use of ECs among pregnant women particularly after marketing efforts have promoted it as a safer alternative to conventional tobacco smoking. However, recent studies have strongly negated this claim and have determined that nicotine in ECs is equally harmful to the fetus and leads to the same degree of cognitive, developmental, neurological, pulmonary, and cardiac decline when compared to the nicotine found in regular tobacco cigarettes (8). This evidence undermines the flawed misconception that ECs are safe to use among pregnant women.

(Appendix A continued)

References used in Appendix A

1. Staudt MR, Salit J, Kaner RJ, Hollmann C, Crystal RG. Altered lung biology of healthy never smokers following acute inhalation of E-cigarettes. Respir Res. 2018;19(1):78; <http://dx.doi.org/doi:10.1186/s12931-018-0778-z>

2. Schober W, Szendrei K, Matzen W, Osiander-Fuchs H, Heitmann D, Schettgen T, et al. Use of electronic cigarettes (e-cigarettes) impairs indoor air quality and increases FeNO levels of e-cigarette consumers. International Journal of Hygiene and Environmental Health. 2014;217(6):628-37; <http://dx.doi.org/doi:10.1016/j.ijheh.2013.11.003>

3. Lødrup Carlsen KC, Skjerven HO, Carlsen K-H. The toxicity of E-cigarettes and children's respiratory health. Paediatric respiratory reviews. 2018:63-7; <http://dx.doi.org/doi:10.1016/j.prrv.2018.01.002>

4. Garcia-Arcos I, Geraghty P, Baumlin N, Campos M, Dabo AJ, Jundi B, et al. Chronic electronic cigarette exposure in mice induces features of COPD in a nicotine-dependent manner. Thorax. 2016;71(12):1119-29; <http://dx.doi.org/doi:10.1136/thoraxjnl-2015-208039>

5. Lee HW, Park SH, Weng MW, Wang HT, Huang WC, Lepor H, et al. E-cigarette smoke damages DNA and reduces repair activity in mouse lung, heart, and bladder as well as in human lung and bladder cells. Proc Natl Acad Sci U S A. 2018;115(7):E1560-E9; <http://dx.doi.org/doi:10.1073/pnas.1718185115>

6. Fuller TW, Acharya AP, Meyyappan T, Yu M, Bhaskar G, Little SR, et al. Comparison of Bladder Carcinogens in the Urine of E-cigarette Users Versus Non E-cigarette Using Controls. Scientific reports. 2018;8(1):507-6; <http://dx.doi.org/doi:10.1038/s41598-017-19030-1>

7. Lippi G, Cervellin G, Favaloro EJ. Response to "Comment on 'E-cigarettes and cardiovascular risk: beyond science and mysticism'". Seminars in thrombosis and hemostasis. 2014;40(4):519-20; <http://dx.doi.org/doi:10.1055/s-0034-1375703>

8. Whittington JR, Simmons PM, Phillips AM, Gammill SK, Cen R, Magann EF, et al. The Use of Electronic Cigarettes in Pregnancy: A Review of the Literature. Obstetrical & Gynecological Survey. 2018;73(9):544-9; <http://dx.doi.org/doi:10.1097/OGX.0000000000000595>

**Appendix B: Validation studies for the questionnaire: one for the translation from English to Arabic and one for the knowledge scale**

In order to ensure the accuracy of the questionnaire’s translation from English to Arabic we carried out a validation study whereby, 26 individuals were approached and asked to fill the questionnaire in both English and Arabic. Our results indicated an internal consistency in the translation with a high overall average percent agreement of 95%. As for the knowledge scale, the internal consistency between the English and Arabic versions of this scale was also assessed in order to validate its translation. Our results, shown in Appendix B, revealed a high and significant agreement in every question. This was determined by the Cohen’s kappa statistic, percent agreement and statistical significance of the agreement. The least agreement was found in the question addressing whether or not nicotine was present in most ECs, where the percent agreement was around 85%, the kappa statistic was 0.72, and the p-value < .0001. However, this level of kappa is still considered very high as per Cohen’s categorization of the levels of agreement. All the remaining questions in the knowledge scale exhibited a high level of internal consistency reaching 100% agreement in some of the questions. This result suggests that the translation, which was done by a licensed translator, was carried out in an accurate manner ensuring the reliability of the translation.

In order to determine the effectiveness of the knowledge scale in identifying good versus poor knowledge, a validation study was carried out. This included a total of 45 participants, 17 of which were experts in ECs and 28 who were not. These 45 individuals were not the same participants who were approached for the validation of translation. The identified experts were nurses and medical doctors specialized in family medicine and pulmonology who have received training and certification in the area of smoking cessation. We targeted non-experts by randomly choosing individuals on the streets of Beirut who had heard of ECs but confirmed that they do not know anything about them. Experts and non-experts filled in the questionnaire individually in the presence of one of the investigators.

Our results revealed that experts had a higher mean knowledge score of 13.18 ±1.131 standard deviation (SD), compared to non-experts who had a mean score of 7.82 ±4.01 SD. The detected increase in the mean knowledge score for experts was statistically significant with an independent t-test p-value < .0001. Along the same line, our non-parametric analysis revealed a higher mean rank for the knowledge score of 33.29 among the experts, compared to a mean rank of 16.75 among non-experts. This increase in mean rank of knowledge score among experts compared to non-experts was statistically significant with a Mann-Whitney test p-value < .0001. Hence, experts had about a two-fold significant increase in the knowledge score compared to non-experts (p-value < .0001). We categorized our validation participants into knowledgeable and not knowledgeable as per our study’s plan of analysis, and compared each participant’s category to their knowledge status (expert vs. non-expert) in order to verify the accuracy of such a classification. Our results revealed that around 88.2% of the experts were correctly classified as knowledgeable, and around 82.1% of the non-experts were correctly classified as non-knowledgeable in ECs. Furthermore, our results showed that the difference in proportion between knowledgeable experts (88.2%) and knowledgeable non-experts (17.9%) was statistically significant with Chi-squared p-value < .0001. Hence, the proportion of experts who were classified as knowledgeable was significantly higher than the proportion of non-experts who were also classified as knowledgeable. These results proved that the knowledge scale was effective in correctly classifying individuals as knowledgeable or not knowledgeable in ECs and thus, confirmed its validity as a knowledge scale.

Appendix B (continued)

| **E-cigarette knowledge questions** | **English questionnaire (ref.)** | | | **Arabic questionnaire** | | | **% Agreement** | | **Kappa value** | | **p-value** |
| --- | --- | --- | --- | --- | --- | --- | --- | --- | --- | --- | --- |
|  | **N** | | **%** | **N** | | **%** |  |  |  |  |  |
| **E-cigarettes are void of harm** | | | | | | | | | | | |
| True | 3 | 11.5 | | 2 | 7.7 | | 96.15 | 0.873 | | .0001 | |
| False | 21 | 80.8 | | 22 | 84.6 | |  |  |  |  |  |
| I do not know | 2 | 7.7 | | 2 | 7.7 | |  |  |  |  |  |
| **E-cigarettes do not contribute to second hand smoking** | | | | | | | | | | | |
| True | 7 | 26.9 | | 7 | 26.9 | | 100 | | 1.000 | | .0001 |
| False | 16 | 61.5 | | 16 | 61.5 | |  |  |  |  |  |
| I do not know | 3 | 11.5 | | 3 | 11.5 | |  |  |  |  |  |
| **E-cigarettes are FDA Approved** | | | | | | | | | | | |
| True | 1 | 3.8 | | 1 | 3.8 | | 100 | | 1.000 | | .0001 |
| False | 12 | 46.2 | | 12 | 46.2 | |  |  |  |  |  |
| I do not know | 13 | 50.0 | | 13 | 50.0 | |  |  |  |  |  |
| **E-cigarettes are suitable for children** | | | | | | | | | | | |
| True | 0 | 0 | | 0 | 0 | | 100 | | 1.000 | | .0001 |
| False | 24 | 92.3 | | 24 | 92.3 | |  |  |  |  |  |
| I do not know | 2 | 7.7 | | 2 | 7.7 | |  |  |  |  |  |
| **E-cigarettes are suitable for pregnant women** | | | | | | | | | | | |
| True | 0 | 0 | | 1 | 3.8 | | 96.15 | | 0.895 | | .0001 |
| False | 20 | 76.9 | | 20 | 76.9 | |  |  |  |  |  |
| I do not know | 6 | 23.1 | | 5 | 19.2 | |  |  |  |  |  |
| **Nicotine is present in most E-cigarettes** | | | | | | | | | | | |
| True | 16 | 61.5 | | 16 | 61.5 | | 84.62 | | 0.717 | | .0001 |
| False | 4 | 15.4 | | 4 | 15.4 | |  |  |  |  |  |
| I do not know | 6 | 23.1 | | 6 | 23.1 | |  |  |  |  |  |
| **E-cigarettes are not addictive** | | | | | | | | | | | |
| True | 7 | 26.9 | | 6 | 23.1 | | 92.31 | | 0.822 | | .0001 |
| False | 19 | 73.1 | | 18 | 69.2 | |  |  |  |  |  |
| I do not know | 0 | 0 | | 2 | 7.7 | |  |  |  |  |  |
| **E-cigarettes can have an effect on fetal development** | | | | | | | | | | | |
| True | 19 | 73.1 | | 20 | 76.9 | | 96.15 | | 0.900 | | .0001 |
| False | 1 | 3.8 | | 0 | 0 | |  |  |  |  |  |
| I do not know | 6 | 23.1 | | 6 | 23.1 | |  |  |  |  |  |
| **Harmful flavorings and toxins are found in the E-cigarette aerosol** | | | | | | | | | | | |
| True | 14 | 53.8 | | 17 | 65.4 | | 88.46 | | 0.788 | | .0001 |
| False | 3 | 11.5 | | 2 | 7.7 | |  |  |  |  |  |
| I do not know | 9 | 34.6 | | 7 | 26.9 | |  |  |  |  |  |
| **Some components of the liquid found in E-cigarettes can cause harmful lung conditions** | | | | | | | | | | | |
| True | 13 | 50.0 | | 14 | 53.8 | | 96.15 | | 0.933 | | .0001 |
| False | 3 | 11.5 | | 2 | 7.7 | |  |  |  |  |  |
| I do not know | 10 | 38.5 | | 10 | 38.5 | |  |  |  |  |  |
| **Swallowing the liquid in E-cigarettes accidentally can cause poisoning that is potentially fatal** | | | | | | | | | | | |
| True | 7 | 26.9 | | 6 | 23.1 | | 92.31 | | 0.877 | | .0001 |
| False | 7 | 26.9 | | 6 | 23.1 | |  |  |  |  |  |
| I do not know | 12 | 46.2 | | 14 | 53.8 | |  |  |  |  |  |
| **E-cigarettes are not associated with lung cancer** | | | | | | | | | | | |
| True | 3 | 11.5 | | 4 | 15.4 | | 92.31 | | 0.871 | | .0001 |
| False | 13 | 50.0 | | 14 | 53.8 | |  |  |  |  |  |
| I do not know | 10 | 38.5 | | 8 | 30.8 | |  |  |  |  |  |
| **E-cigarettes are associated with bladder cancer** | | | | | | | | | | | |
| True | 9 | 34.6 | | 9 | 34.6 | | 88.46 | | 0.814 | | .0001 |
| False | 5 | 19.2 | | 4 | 15.4 | |  |  |  |  |  |
| I do not know | 12 | 46.2 | | 13 | 50.0 | |  |  |  |  |  |
| **E-cigarettes are associated with heart disease** | | | | | | | | | | | |
| True | 14 | 53.8 | | 15 | 57.7 | | 92.31 | | 0.866 | | .0001 |
| False | 4 | 15.4 | | 2 | 7.7 | |  |  |  |  |  |
| I do not know | 8 | 30.8 | | 9 | 34.6 | |  |  |  |  |  |
| **E-cigarettes could impair lung function** | | | | | | | | | | | |
| True | 20 | 76.9 | | 19 | 73.1 | | 96.15 | | 0.900 | | .0001 |
| False | 0 | 0 | | 1 | 3.8 | |  |  |  |  |  |
| I do not know | 6 | 23.1 | | 6 | 23.1 | |  |  |  |  |  |
| **Some flavors of E-cigarettes are more harmful than others** | | | | | | | | | | | |
| True | 10 | 38.5 | | 9 | 34.6 | | 92.31 | | 0.884 | | .0001 |
| False | 8 | 30.8 | | 7 | 26.9 | |  |  |  |  |  |

Appendix B (continued)

Appendix C: Bivariate analysis of the outcome attitude towards E-cigarettes with its covariates ^τ^

| **Covariate** | | **No. (%)** | **Unadjusted Odds Ratio** | **95% Confidence Interval for Odds Ratio** | | **P-value^ŧ^** |
| --- | --- | --- | --- | --- | --- | --- |
|  |  |  |  | **Lower Limit** | **Upper Limit** |  |
| Age  Mean ± SD | | 30.3 ± 11.8 | .965 | .919 | 1.013 | .150 |
| Sex | Male (Reference) | 198 (56.6) | .464 | .188 | 1.143 | .095 |
|  | Female | 152 (43.4) |  |  |  |  |
| Heard about E-cigarettes from social media | | 172  (54.1) | .527 | .221 | 1.256 | .148 |
| Heard about E-cigarettes from family | | 84  (26.5) | .398 | .115 | 1.374 | .145 |
| Use E-cigarettes | | 36  (10.9) | 3.056 | 1.127 | 8.288 | .028* |
| Drink coffee | | 281  (80.5) | .516 | .204 | 1.306 | .163 |
| Reason for not using E-cigarettes:  Harmful/ unhealthy | | 73  (25.9) | .158 | .021 | 1.211 | .076 |
| E-cigarettes are harmful | | 215  (65.3) | .269 | .115 | .630 | .002* |
| E-cigarettes contribute to second hand smoking | | 178  (53.8) | .379 | .159 | .904 | .029* |
| E-cigarettes are addictive | | 223  (67.0) | .205 | .085 | .491 | .000* |
| E-cigarettes impair lung and heart function | | 216 (66.3) | .255 | .109 | .599 | .002* |
| E-cigarettes are not suitable for pregnant women | | 298  (89.5) | .433 | .152 | 1.238 | .118 |
| E-cigarettes can have an effect on fetal development | | 201  (60.4) | .491 | .216 | 1.117 | .090 |
| Harmful flavorings and toxins are found in the E-cigarette aerosol | | 204  (62.0) | .212 | .086 | .524 | .001* |
| Some components of the liquid found in E-cigarettes can cause harmful lung conditions | | 220  (66.5) | .306 | .133 | .706 | .006* |
| Swallowing the liquid in E-cigarettes accidentally can cause poisoning that is potentially fatal | | 148  (44.7) | .367 | .143 | .945 | .038* |
| E-cigarettes are associated with bladder cancer | | 82  (24.6) | .400 | .117 | 1.374 | .146 |

* p-value ≤ .050  ^τ^ covariates also included smoking cigars (p-value = .045) and quitting E-cigarettes (p- value = .023) but data is not shown due to small sample size in these covariates.

Appendix C (continued)

^ŧ^ Variables with p-value ≤ .200 have been deemed eligible to enter the multivariable model.

Appendix D: Bivariate analysis of the outcome knowledge of E-cigarettes with its covariates

| **Covariate** | **No. (%)** | **Unadjusted Odds Ratio** | **95% Confidence Interval for Odds Ratio** | | **P-value^ŧ^** |
| --- | --- | --- | --- | --- | --- |
|  |  |  | **Lower Limit** | **Upper Limit** |  |
| Level of Education: Technical/Vocational | 7 (2.0) | .687 | .402 | 1.175 | .17 |
| Health Exposure in Occupation/Major | 64 (21.9) | 1.729 | .974 | 3.071 | .061 |
| Smokers | 162 (46.2) | .591 | .363 | .961 | .034* |
| Quit/Process of Quitting Smoking | 38 (10.8) | .562 | .245 | 1.291 | .174 |
| Exercise regularly | 155 (44.0) | 1.691 | 1.065 | 2.684 | .026* |
| Heard about E-Cigarettes in Center for Smoking Cessation | 9 (2.8) | 4.265 | 1.045 | 17.410 | .043* |
| Use E-Cigarettes | 36 (10.9) | .475 | .201 | 1.124 | .090 |
| Smoke: Hookah | 68 (37.8) | .607 | .290 | 1.270 | .185 |
| Use E-Cigarettes as Smoking Cessation Method | 12 (15.0) | .174 | .021 | 1.433 | .104 |
| Use Other Methods as Smoking Cessation Method | 29 (35.4) | .383 | .124 | 1.179 | .094 |
| Reducing the Number of Cigarettes/Day as Smoking Cessation Method | 39 (48.1) | 2.097 | .774 | 5.677 | .145 |
| Reason for Not Using E-Cigarettes: Never Considered It | 118 (41.7) | .691 | .414 | 1.154 | .158 |
| Reason for Not Using E-Cigarettes: Harmful/Unhealthy | 73 (25.9) | 1.694 | .973 | 2.949 | .063 |
| Think the government should not regulate the use of E-Cigarettes | 97 (30.0) | .704 | .418 | 1.185 | .187 |
| Think that the use of E-Cigarettes should be allowed in places that do not allow smoking | 66 (20.3) | .345 | .172 | .692 | .003* |
| Feel more comfortable using or openly talking about smoking E-Cigarettes, compared to cigarettes | 97 (30.9) | .438 | .251 | .762 | .004* |
| Think using E-Cigarettes would be an effective way to help in smoking cessation | 156 (48.6) | .448 | .278 | .723 | .001* |
| Think E-Cigarettes should be used as a replacement for regular cigarettes | 143 (44.3) | .517 | .319 | .836 | .007* |
| Think E-Cigarettes should be recommended to a nonsmoker | 32  (9.7) | .554 | .232 | 1.326 | .185 |
| Think E-Cigarettes are not harmful for health | 51 (15.8) | .142 | .050 | .405 | .000* |
| Think the use of E-Cigarettes does not lead to reliance | 75 (23.6) | .370 | .196 | .699 | .002* |
| Think it is acceptable to experiment with E-Cigarettes for pleasure | 145 (44.3) | .528 | .327 | .855 | .009* |
| Consider someone who uses E-Cigarettes as a non-smoker | 75 (22.9) | .578 | .321 | 1.043 | .069 |

Appendix D (continued)

* p-value ≤ .050

^ŧ^ Variables with p-value ≤ .200 have been deemed eligible to enter the multivariable model.
